# Average CT in PET studies of colorectal cancer patients with metastasis in the liver and esophageal cancer patients

**DOI:** 10.1120/jacmp.v11i1.3073

**Published:** 2010-02-04

**Authors:** Elena Tonkopi, Pai‐Chun Melinda Chi, Osama Mawlawi, Adam C. Riegel, Eric M. Rohren, Homer A. Macapinlac, Tinsu Pan

**Affiliations:** ^1^ Department of Imaging Physics The University of Texas M. D. Anderson Cancer Center Houston TX USA; ^2^ Department of Radiation physics The University of Texas M. D. Anderson Cancer Center Houston TX USA; ^3^ Department of Nuclear Medicine The University of Texas M. D. Anderson Cancer Center Houston TX USA

**Keywords:** respiration‐averaged CT, PET/CT, respiration‐induced artifact

## Abstract

Average CT (ACT) and PET have a similar temporal resolution and it has been shown to improve registration of the CT and PET data for PET/CT imaging of the thorax. The purpose of this study was to quantify the effect of ACT attenuation correction on PET for gross tumor volume (GTV) delineation with standardized uptake value (SUV) for liver and esophageal lesions. Our study included 48 colorectal cancer patients with metastasis in the liver and 52 esophageal cancer patients. These patients underwent a routine PET/CT scan followed by a cine CT scan of the thoracic region for ACT. Differences between the two PET data sets (${\text{PET}}_{\text{HCT}}$ and ${\text{PET}}_{\text{ACT}}$) corrected with the helical CT (HCT) and ACT were quantified by analyzing image alignment, maximum SUV $\left({\text{SUV}}_{\max}\right)$, and GTV. The 67% of the colorectal and 73% of the esophageal studies demonstrated misregistration between the ${\text{PET}}_{\text{HCT}}$ and HCT data. ACT was effective in removing misregistration artifacts in 65% of the misregisted colorectal and in 76% of the misregisted esophageal cancer patients. Misregistration between the CT and PET data affected GTVs due to the change in ${\text{SUV}}_{\max}$ with ACT. A change of ${\text{SUV}}_{\max}$ greater than 20% between ${\text{PET}}_{\text{HCT}}$ and ${\text{PET}}_{\text{ACT}}$ was found in 15% of the colorectal and 17% of the esophageal cases. Our results demonstrated a more pronounced effect of misregistration for the smaller lesions ${(<5\text{cm}}^{3})$ near the diaphragm (<5cm). ACT was effective in improving registration between the CT and PET data in PET/CT for the colorectal and esophageal cancer patients.

PACS number: 87.58.Fg

## I. INTRODUCTION

The emergence of positron emission tomography/computed tomography (PET/CT) made it possible to provide anatomic and functional information in a single examination and to improve the sensitivity and specificity of tumor detection over PET or CT alone.^(^ ^1^ ^–^ ^3^ ^)^ PET/CT imaging is now part of routine clinical practice in oncology for diagnosis, staging, and monitoring of tumor response to therapy.^(^ ^4^ ^–^ ^6^ ^)^ PET/CT has also gained acceptance in cardiology for coronary artery imaging and myocardial functional assessment.^(^ ^7^ ^–^ ^13^ ^)^ Standardized uptake value (SUV) is normally used as an indicator of malignancy.^(^ ^14^ ^,^ ^15^ ^)^ Over 50% of all patients with cancer receive radiation therapy and FDG‐PET has been shown to influence the decision of target volumes for non‐small–cell lung cancers (NSCLC), esophageal tumors, and head‐and‐neck squamous cell carcinomas.^(^ ^16^ ^–^ ^24^ ^)^ Incorporation of PET data into treatment volume delineation for NSCLC can increase or decrease the treated volume between 15% to 60%.^(19)^ Since CT data is used for attenuation correction (AC) of PET data, spatial misregistration between the CT and PET data may compromise the quantification of PET. Respiratory motion during imaging – in combination with the difference in temporal resolution between the PET and CT data – could cause misregistration between the PET and CT data, compromising the advantages of this hybrid imaging modality.^(25)^ This misregistration problem did not exist in the stand‐alone PET scanners, which used transmission rod sources to acquire transmission data for AC. However, stand‐alone PET scanners suffered from a long transmission scan time. As a result, most patients were scanned in the position of arms down, different from the arms up position typically used in the radiotherapy simulation or treatment, making it more complex in registration of the PET and CT data. In addition, the PET and CT data were acquired as two different sessions and at two different times. Issues pertaining to software registration of the PET and CT data acquired in two different scanners have been reduced significantly with hardware registration of the PET/CT scanner.^(26)^

To address the registration problem from respiration in PET/CT, there are multiple studies investigating different approaches to correct respiratory motion artifacts. Some investigated different CT scanning protocols^(^ ^25^ ^,^ ^27^ ^–^ ^41^ ^)^ including deep inspiration breath hold,^(^ ^30^ ^–^ ^32^ ^)^ mid‐expiration breath‐hold,^(42)^ shallow breathing,^(^ ^33^ ^,^ ^43^ ^)^ respiratory gating,^(^ ^35^ ^–^ ^39^ ^,^ ^41^ ^)^ or using a slow CT scan.^(40)^ Each of the recommendations has its advantages and disadvantages. For instance, complying with the breathing protocol can be difficult for some patients.^(44)^ Gated PET images usually exhibit reduced statistics; therefore, they need a longer acquisition time.^(45)^ Slow CT protocol is unreliable in depicting the average motion of respiration.^(46)^ AC of the PET images with CT data on phase‐to‐phase basis requires 4D acquisition of both PET and CT, which may not be practical.

We have proposed the use of respiration‐averaged CT (ACT) to match the temporal resolution of CT and PET, and have shown that ACT reduces respiratory artifacts in PET/CT.^(^ ^46^ ^,^ ^47^ ^)^ ACT acquisition has been optimized for clinical implementation and is available on the PET/CT scanners in our institution. In order to quantify the effect of ACT, data was acquired under the institution review board protocol DR07‐0560 for three months and results have been analyzed. An extensive analysis on the 229 lung cancer patients^(48)^ has shown an improvement of the PET and CT data registration with the ACT technique and its potential effect on GTV delineation. The other anatomical areas prone to respiratory induced artifacts are the esophagus and the upper abdomen,^(^ ^49^ ^–^ ^51^ ^)^ and this study focused on these particular regions. The aim of our investigation was to demonstrate a potential improvement in the PET/CT data registration and the effect on GTV delineation by the usage of ACT in patients with esophageal cancer or colorectal cancer with metastasis in the liver.

## II. MATERIALS AND METHODS

### A. Data acquisition

Our study included 48 colorectal cancer patients with metastasis in the liver and 52 esophageal cancer patients. These patients had undergone a routine PET/CT examination for diagnosis or post‐therapy evaluation. All the data were acquired on three different PET/CT scanners (DST/RX/DSTE; GE Healthcare, Waukesha, WI). The patients were injected with 555‐740 MBq of F18‐FDG and scanned 1 h after injection. A helical CT (HCT) scan was acquired in 16 and 32 sec over 90 cm coverage by a 16‐slice and 8‐slice CT, respectively, followed by a 2D PET scan of 3 min per bed and a cine CT scan of the thorax (approximately 20 cm) for ACT. Patients were free breathing during all scans without any coaching of respiration. Both HCT and ACT were used for AC of the PET data, resulting in two data sets: PET corrected with HCT $\left({\text{PET}}_{\text{HCT}}\right)$ and PET corrected with ACT $\left({\text{PET}}_{\text{ACT}}\right)$.

HCT data were acquired at 120 kVp, 300 mA, 1.35 pitch for 8‐slice and 1.375 pitch for 16‐slice, and 0.5 sec gantry rotation. X‐ray collimation was 8×1.25mm on the 8‐slice DST and 16×1.25mm on the 16‐slice RX and DSTE. Cine duration was 5.9 sec per 2 cm coverage for capturing at least one respiratory cycle.^(^ ^46^ ^,^ ^47^ ^,^ ^52^ ^)^ The other parameters for cine scan were 120 kVp and collimation of 8×2.5mm for both 8‐ and 16‐slice CTs. Tube current was adjusted according to the patients’ weight: 10 mA for <70kg, 15 mA for 70‐100 kg, and 20 mA for <100kg. Processing time of ACT for PET attenuation correction was less than 5 min; additional radiation dose from the cine CT scan for ACT was 5–10 mGy^(46)^ or 1.7–3.4 mSv.

### B. Data analysis

**Figure 1:**
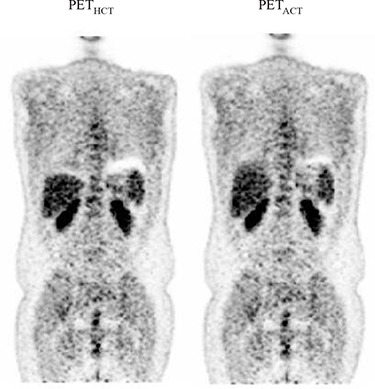
The ${\text{PET}}_{\text{HCT}}$ (left) and ${\text{PET}}_{\text{ACT}}$ images (right) of a patient study. The misalignment at the diaphragm level was measured as the thickness of a photopenic region or a white band. The artifact was reduced in the ${\text{PET}}_{\text{ACT}}$.

The method described in our previous study of the lung cancer patients^(48)^ was adopted for data analysis. The ${\text{PET}}_{\text{HCT}}$ and ${\text{PET}}_{\text{ACT}}$ images were compared in quantitation using viewing software (Advantage Workstation (AW) 4.2, GE Healthcare, Waukesha, WI). Misalignment between the CT and PET data due to respiratory motion was measured as the thickness of a photopenic region or white band at the diaphragm level. An example is shown in Fig. 1. Another criterion to quantify the difference was ${\text{SUV}}_{\max}$, measured on both ${\text{PET}}_{\text{HCT}}$ and ${\text{PET}}_{\text{ACT}}$ with the AW workstation. The percent changes in ${\text{SUV}}_{\max}$ were calculated in reference to ${\text{PET}}_{\text{HCT}}$.

Both PET data sets were analyzed on a commercial treatment planning system (${\text{Pinnacle}}^{3}$ 7.6, Philips Medical Systems, Andover, MA). The images were displayed in SUV using a software tool developed in‐house. The gross tumor volumes (GTVs) were generated based on the SUV thresholds. The initial threshold for the delineation was 2.5, which usually indicates potential malignancy of a lesion.^(53)^ In some cases when the SUV was too close to the background uptake, the threshold had to be increased until the GTV delineation could be performed by SUV threshold. Typical values were 3.0–3.5 for the colorectal and 3.0–3.3 for the esophageal lesions. The same SUV threshold was chosen for both ${\text{PET}}_{\text{HCT}}$ and ${\text{PET}}_{\text{ACT}}$ images of the same patient. In this study, the following parameters were analyzed: the centroid shift between the GTVs corresponding to the ${\text{PET}}_{\text{HCT}}$ and ${\text{PET}}_{\text{ACT}}$, the percent change in the GTV volume, and the concordance index. The centroid location and the GTV volume were measured. The concordance index was defined as the intersection of the two GTVs corresponding to the ${\text{PET}}_{\text{HCT}}$ and ${\text{PET}}_{\text{ACT}}$ divided by their union,^(54)^ and it was calculated using a MATLAB program written to read the regions of interest defined in the treatment planning system. Colorectal and esophageal cancer studies were analyzed and presented separately.

## III. RESULTS

### A. Respiratory artifacts evaluation and ${\text{SUV}}_{\max}$ comparison

**Figure 2:**
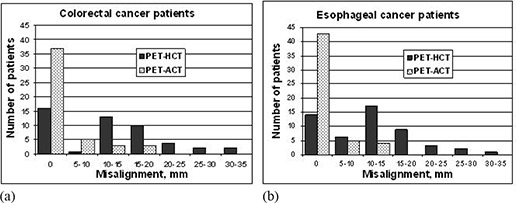
Two histograms showing the misalignment between ${\text{PET}}_{\text{HCT}}$ and ${\text{PET}}_{\text{ACT}}$: (a) 67% of the 48 colorectal and (b) 73% of the 52 esophageal cancer patients demonstrated misalignment of over 5 mm between the CT and PET data with maximum magnitude of 33 mm for the colorectal and 31 mm for the esophageal cancers. There was less misregistration in ${\text{PET}}_{\text{ACT}}$ than in ${\text{PET}}_{\text{HCT}}$.

On ${\text{PET}}_{\text{HCT}}$ images, 32 colorectal (67%) and 38 esophageal (73%) cancer patients demonstrated the photopenic region or white band artifacts as a result of mismatch between the PET and HCT data. ${\text{PET}}_{\text{ACT}}$ was effective in removing this artifact for 21 colorectal and for 29 esophageal cancer patients. In those cases when the misalignment was not completely removed, ${\text{PET}}_{\text{ACT}}$ was still effective in reducing it. The results of the comparison between ${\text{PET}}_{\text{HCT}}$ and ${\text{PET}}_{\text{ACT}}$ are shown in Fig. 2. The mean values of the misalignment on ${\text{PET}}_{\text{HCT}}$ for the colorectal and esophageal patients were respectively 16±6mm and 14±5mm, with the maximum magnitude up to 33 mm for one colorectal patient. The mean values of the misalignment on ${\text{PET}}_{\text{ACT}}$ for the cases when it was not completely removed were 11±4mm for the colorectal and 10±2mm for the esophageal patients.

**Figure 3:**
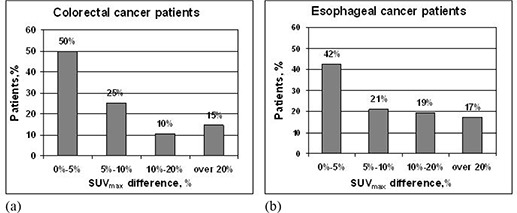
Two histograms showing the difference in ${\text{SUV}}_{\max}$ between ${\text{PET}}_{\text{HCT}}$ and ${\text{PET}}_{\text{ACT}}$: (a) 15% of the colorectal cancer patients and (b) 17% of the esophageal cancer patients showed a difference in ${\text{SUV}}_{\max}$ of over 20%.

In the analysis of ${\text{SUV}}_{\max}$ change for the patients with multiple lesions only the lesion with the highest percentage change in the ${\text{SUV}}_{\max}$ was included. Therefore, there were 48 colorectal and 52 esophageal lesions considered. Figure 3 shows the results of the ${\text{SUV}}_{\max}$ change. There were 15% of the colorectal and 17% of the esophageal cancer patient studies demonstrating a percent change of over 20% in ${\text{SUV}}_{\max}$, with the most extreme cases of an esophageal lesion showing 50% increase and a liver lesion showing 29% increase in ${\text{SUV}}_{\max}$ on ${\text{PET}}_{\text{ACT}}$ data. In the majority of cases, a higher ${\text{SUV}}_{\max}$ value was measured on ${\text{PET}}_{\text{ACT}}$; however, in five esophageal patient studies, ${\text{SUV}}_{\max}$ was higher on ${\text{PET}}_{\text{HCT}}$. Absolute values of the percent change were included in the analysis.

### B. GTVs analysis in Pinnacle

**Figure 4:**
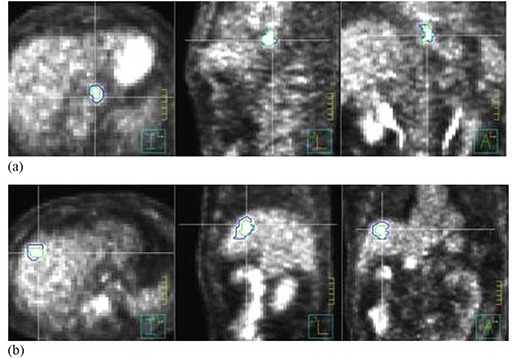
Examples of segmented GTVs on transverse, sagittal, and coronal views of the ${\text{PET}}_{\text{ACT}}$ images: (a) the liver lesion of a colorectal cancer patient and (b) an esophageal tumor. The green contour is generated from ${\text{PET}}_{\text{HCT}}$ and the blue contour is generated from ${\text{PET}}_{\text{ACT}}$.

**Figure 5:**
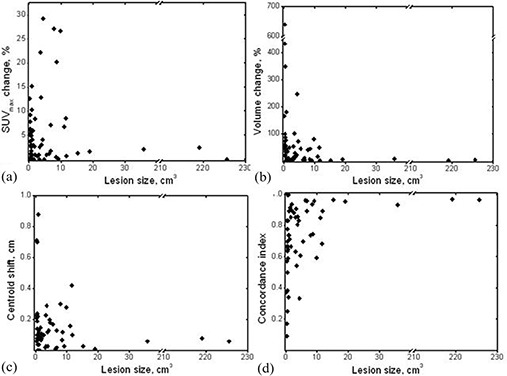
Scatter plots for (a) percent ${\text{SUV}}_{\max}$ change, (b) GTV volume change, (c) centroid shift, and (d) concordance index for the colorectal cancer patients between ${\text{PET}}_{\text{HCT}}$ and ${\text{PET}}_{\text{ACT}}$.

**Figure 6:**
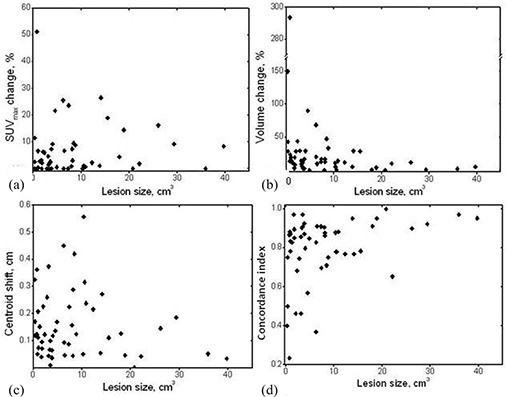
Scatter plots for (a) percent ${\text{SUV}}_{\max}$ change, (b) GTV volume change, (c) centroid shift, and (d) concordance index for the esophageal cancer patients between ${\text{PET}}_{\text{HCT}}$ and ${\text{PET}}_{\text{ACT}}$. All lesions were less than 50 cm^3^.

Proper GTV delineation could not be performed for 13 colorectal and 6 esophageal cancer patients due to low tumor‐to‐background ratio. Those patients were excluded from the GTV analysis. Some patients had multiple lesions. Overall, there were 52 esophageal and 59 liver lesions included in the study. Figure 4 shows the transverse, sagittal, and coronal views of the ${\text{PET}}_{\text{ACT}}$ images for two patients: one from the colorectal cancer group and the other one from the esophageal cancer group. The green contour was generated from ${\text{PET}}_{\text{HCT}}$ and the blue contour was generated from ${\text{PET}}_{\text{ACT}}$. For the first example with a liver lesion, the percent ${\text{SUV}}_{\max}$ change was 29%, volume change was 246% with an increase in absolute value of 10.6 cm^3^, centroid shift was 2 mm and concordance index was 0.336. For the second example with an esophageal lesion, the percent ${\text{SUV}}_{\max}$ change was 22%, volume change was 90% with an increase in absolute value of 4.1 cm^3^, centroid shift was 14 mm and concordance index was 0.569. The scatter plots of the parameter changes versus lesion size are shown in Fig. 5 for the colorectal cancer and in Fig. 6 for the esophageal cancer patients. Both patient data sets demonstrate a more pronounced effect of misalignment for the smaller lesions. For instance, for the liver lesions, the highest percent volume change of 640% corresponds to the 1.6 cm^3^ absolute change of the lesion size in ${\text{PET}}_{\text{HCT}}$ of 0.25 cm^3^. The same lesion shows the smallest concordance index of 0.09 in the colorectal patient population, and one of the largest changes in the centroid shift of 7.1 mm. The same trend was also noticed for the esophageal lesions. The highest percent volume change of 294% corresponding to the 2.3 cm^3^ change of the lesion size in ${\text{PET}}_{\text{HCT}}$ of 0.78 cm^3^ was found for the lesion with the lowest concordance index of 0.23, and with one of the highest centroid shift of 3.6 mm.

### C. Correlation with the lesion size and location

The liver and esophageal lesions were smaller in size than the lung lesions in our previous study.^(48)^ In current study, the average liver lesion was 10.8 cm^3^ and the average esophageal lesion was 8.5 cm^3^. The criterion to define “small” and “large” lesions was chosen to be 5 cm^3^, which is approximately half of the average size for both patient data sets. The mean values of the analyzed parameters for those distributions are shown in Table 1. A two‐tailed, nonpaired t‐test was used to evaluate the difference between the small and the large lesions. The results for both sets of patients showed that at p‐value 0.05, the difference is not significant for the means of the following parameters: percent ${\text{SUV}}_{\max}$ change (p=0.392 for the liver and p=0.781 for the esophageal lesions), percent volume change (p=0.068 for the liver, p=0.057 for the esophageal lesions), and centroid shift (p=0.456 for the liver, p=0.864 for the esophageal lesions). The only significant difference was found for the concordance index for both patient data sets: p=0.005 for the liver lesions and p=0.018 for the esophageal lesions.

**Table 1:** Distribution of the parameters according to the lesion size.

*Patients*	*Lesion Volume*	*Number of Lesions*	*Mean* $\Delta {\text{SUV}}_{\max},\;\%$	*Mean ΔVolume, %*	*Mean Centroid Shift, cm*	*Mean Concordance Index*
Colorectal Cancer	$<5\;{\text{cm}}^{3}$	43	5	76.6	0.16	0.70
	$>5\;{\text{cm}}^{3}$	16	7	17.4	0.12	0.91
Esophageal Cancer	$<5\;{\text{cm}}^{3}$	27	6	36.3	0.15	0.74
	$>5\;{\text{cm}}^{3}$	25	7	12.3	0.16	0.85

**Figure 7:**
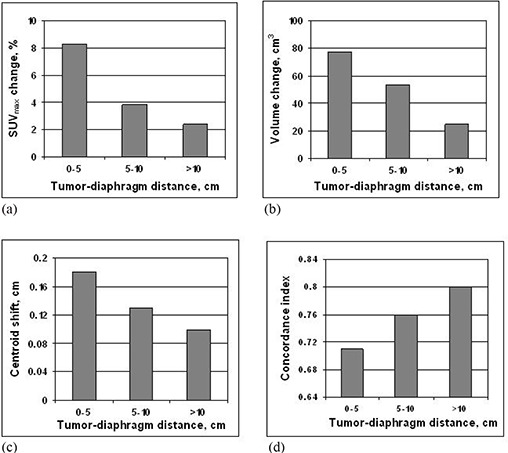
Mean values of (a) percent ${\text{SUV}}_{\max}$ change, (b) GTV volume change, (c) centroid shift, and (d) concordance index for each group of the liver lesions between ${\text{PET}}_{\text{HCT}}$ and ${\text{PET}}_{\text{ACT}}$, defined according to their distance to the diaphragm.

**Figure 8:**
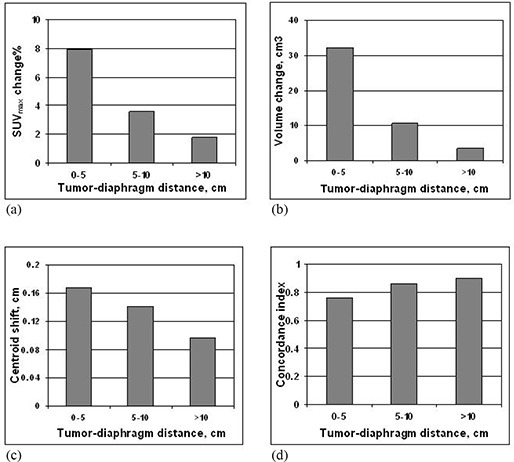
Mean values of (a) percent ${\text{SUV}}_{\max}$ change, (b) GTV volume change, (c) centroid shift, and (d) concordance index for each group of the esophageal lesions between ${\text{PET}}_{\text{HCT}}$ and ${\text{PET}}_{\text{ACT}}$, defined according to their distance to the diaphragm.

To analyze correlation with lesion location, all lesions were divided in three groups according to the absolute distance from the diaphragm: 0<d<5cm,5cm<d<10cm, and d>10cm, as shown in Table 2. The maximum distance 16.6 cm was measured for the esophageal lesions, located superior to the diaphragm. A one‐way ANOVA test at 0.05 p‐value level was used to determine any difference between those groups. The only significant difference between variations in different groups (p=0.04) was found for $\Delta {\text{SUV}}_{\text{max}}$ for the liver lesions. Figures 7 and 8 demonstrate the mean values of percent ${\text{SUV}}_{\max}$ change, percent volume change, centroid shift, and concordance index for each group of the liver and esophageal lesions.

**Table 2:** Distribution of the parameters according to the lesion location.

*Patients*	*Lesion‐Diaphragm Distance, cm*	*Number of Lesions*	*Mean* $\Delta {\text{SUV}}_{\max},\;\%$	*Mean ΔVolume, %*	*Mean Centroid Shift, cm*	*Mean Concordance Index*
Colorectal Cancer	0<d<5	23	8	77.6	0.18	0.71
	5<d<10	31	4	53.6	0.13	0.76
	d>10	5	2	24.7	0.10	0.80
Esophageal Cancer	0<d<5	37	8	32.3	0.17	0.76
	5<d<10	8	4	10.6	0.14	0.86
	d>10	7	2	3.5	0.10	0.90

## IV. DISCUSSION

The influence of respiratory motion in PET/CT on liver and esophageal lesions was investigated. The PET data of 48 colorectal cancer patients with metastasis in the liver and 52 esophageal cancer patients were attenuation corrected with both HCT and ACT. Respiratory artifacts were present in ${\text{PET}}_{\text{HCT}}$ data in 67% of the colorectal and 73% of the esophageal patients. ACT was effective in removing misregistration artifacts in 65% of the misregisted colorectal and 76% of the misregisted esophageal cancer patients. Another parameter used for quantification of the differences between the two methods was ${\text{SUV}}_{\max}$ value, commonly used in PET imaging for the treatment response monitoring and the baseline evaluation of the patients. A reduction of at least 15 – 25% in tumor uptake after one cycle of chemotherapy is classified as a partial metabolic response.^(^ ^55^ ^,^ ^56^ ^)^ Variations in ${\text{SUV}}_{\max}$ of over 20% have been found in 15% of the colorectal and in 17% of the esophageal patient populations. Therefore, under‐estimation as well as over‐estimation of the ${\text{SUV}}_{\max}$ due to misregistration of the PET and CT data could potentially change the patient management. These results are consistent with the results reported in our previous study of the lung cancer patients.^(48)^

Treatment planning based on PET/CT could also be affected by misalignment between the PET and CT data. It was shown in this study that localization of the tumor and delineation of the GTVs could be impacted by the use of ACT. Since ACT has a better registration with PET data than HCT, as it reduced misregistration, the GTV generated on ${\text{PET}}_{\text{ACT}}$ image is expected to be more accurate. The changes influenced by misregistration are dependent on the lesion size and location: the smaller lesions located closer to the diaphragm were typically affected more by misregistration. This result is similar to the conclusion of our previous investigation of lung patients.^(48)^ However, there were some differences in the two studies due to the different anatomic regions. The average liver lesion was 10.8 cm^3^ and the average esophageal lesion was 8.5 cm^3^, therefore our criterion for the definition of a small lesion was chosen to be less than 5 cm^3^. It was 50 cm^3^ for the lung lesions.^(48)^ Both the liver and esophageal lesions showed a significant difference between the “small” and “large” groups only for concordance index, the lung lesions also demonstrated a significant difference in percent ${\text{SUV}}_{\max}$ change. For the lung cancer patients, the correlation between the variations and the lesion locations showed a significant difference for the tumors located below the dome of the diaphragm (d≤0). In this study the absolute distance from the diaphragm was chosen as a criterion because all the liver lesions are located inferior to the diaphragm level. Three different groups (0<d<5,5<d<10,d>10cm) were considered and the only significant difference was found to be the percent change in ${\text{SUV}}_{\max}$ for the liver lesions. There was the same general trend in both studies that the parameter variation decreases as tumor‐diaphragm distance increases. Overall the liver and the esophageal lesions demonstrated less dependence on the respiratory artifacts, because the majority of those lesions are located further away from the diaphragm level than the lung lesions.

## V. CONCLUSIONS

Our study demonstrated that using ACT for attenuation correction of the PET data could successfully reduce misregistration between the PET and CT data due to respiratory motion. The respiration artifacts can affect ${\text{SUV}}_{\max}$ values and segmented GTVs in delineation of the esophagus and the liver lesions, having a potential to change the patient's management and treatment. Smaller lesions located near the diaphragm are affected the most by respiratory motion. Based on our previous and current studies, ACT has become part of a standard clinical protocol in our institution for PET/CT acquisition for treatment planning of the lung and esophageal cancer patients.

## ACKNOWLEDGEMENTS

The authors would like to thank Rebecca Marsh, Ph.D. for the very helpful comments on this paper.
